# *Histoplasma capsulatum *yeast phase-specific protein Yps3p induces Toll-like receptor 2 signaling

**DOI:** 10.1186/1742-2094-5-30

**Published:** 2008-07-07

**Authors:** Rajagopal N Aravalli, Shuxian Hu, Jon P Woods, James R Lokensgard

**Affiliations:** 1 Center for Infectious Diseases and Microbiology Translational Research, University of Minnesota Medical School, Minneapolis, Minnesota, USA; 2 Department of Medical Microbiology and Immunology, University of Wisconsin Medical School, Madison, Wisconsin, USA; 3 Department of Radiology, Mayo MMC 292, University of Minnesota Medical School, 420 Delaware Street SE, Minneapolis, MN 55455, USA

## Abstract

*Histoplasma capsulatum *is a common cause of fungal infection in certain geographic areas, and although most infections are asymptomatic, it is capable of causing histoplasmosis, a disseminated, life-threatening disease, especially in immunocompromised individuals. A deeper understanding of this host-pathogen interaction is needed to develop novel therapeutic strategies to counter lethal infection. Although several lines of evidence suggest that this fungus is neurotropic in HIV patients, little is known about the immunobiology of *Histoplasma *infection in the central nervous system [CNS]. The goal of the present study was to understand the innate neuroimmune mechanisms that recognize *H. capsulatum *during the initial stages of infection. Using a 293T stable cell line expressing murine Toll-like receptor 2 [TLR2], we show here that TLR2 recognizes *H. capsulatum *cell wall protein Yps3p and induces the activation of NF-κB. In further experiments, we tested the ability of Yps3p to induce signaling from TLR2 in primary microglial cells, the resident brain macrophages of the CNS. Our data show that *H. capsulatum *Yps3p induced TLR2 signaling in wild-type microglia, but not in microglia isolated from TLR2 KO mice, confirming that Yps3p is a ligand for TLR2. Furthermore, Yps3p-induced TLR2 signaling was suppressed by vaccinia virus-encoded TLR inhibitors. This is the first demonstration of a fungal protein serving as a TLR ligand and mediating signaling in primary brain cells.

## Background

Inhalation of the human pathogenic fungus *Histoplasma capsulatum *may result in histoplasmosis, an important emerging infectious disease that occurs in immunocompromised individuals and transplant patients [1]. Among the known varieties of this opportunistic fungus, *H. capsulatum *var *capsulatum *[referred hereafter as *H. capsulatum*] is present mostly in North and Central America, whereas *H. capsulatum *var. *duboisii *is endemic in Africa (reviewed in [2]). Histoplasmosis has also been reported to occur in the central nervous system [CNS] [1,3-5]. Current treatments for CNS histoplasmosis with amphotericin B combined with one of two commonly used azoles, fluoconazole and itraconazole, have not been encouraging [2,6-8] although successful outcomes have been reported [7,9,10]. In some instances, histoplasmosis may manifest either as myelopathy or as brain tumor further complicating the diagnosis [11,12]. Treatment with fluconazole in the mouse model of intracranial infection has been proved to be ineffective [6]. Therefore, extensive efforts are being made to develop novel diagnostic tools and anti-fungal therapies to diagnose histoplasmosis and to curtail its progression.

*H. capsulatum *is a dimorphic fungus that exists as mycelium at 25°C and as yeast at 37°C [13]. Conversion of mycelium to the yeast phase has been demonstrated to be critical for pathogenicity of the fungus as agents that inhibit the dimorphic transition, such as *p*-chloromercuriphenylsulfonic acid, render virulent *H. capsulatum *strains avirulent [14]. Macrophages provide a protected environment for *H. capsulatum *to multiply and disseminate from the lungs to other organs. Initial studies with murine macrophages demonstrated that *H. capsulatum *could survive in the harsh conditions of phagolysosomal compartments [15] and modulate the pH of its intracellular niche [16]. This fungus was later shown to survive in 'modified' lysosomes in human macrophages, as well in the RAW264.7 cell line [17].

Toll-like receptors [TLRs] are a class of pathogen-recognition receptors that recognize specific molecular patterns [PAMPs] on the surface of invading pathogens and generate innate immune responses to counter infection [18]. Microglia have been shown to express mRNAs for all known TLRs [19], and recent reports demonstrate that TLR2 on microglial cells recognizes a number of PAMPs and triggers immune responses [20-22]. A critical role for TLRs in recognizing and triggering innate immune responses against several opportunistic fungal pathogens such as *Candida albicans*, *Aspergillus fumigatus*, and *Cryptococcus neoformans *have been reported [23-32]. In contrast to these organisms, little is known about the involvement of TLRs in host responses to dimorphic fungi such as *H. capsulatum*, *Coccidiodes immitis*, *Blastomyces dermatitidis *and *Paracoccoidioides brasiliensis*. To date, fungal cell wall and capsule components such as phospholipomannan and zymosan were reported to be ligands for a number of cellular receptors, including the TLRs, but specific fungal proteins that could induce signaling from these receptors have not yet been identified.

Several *H. capsulatum *genes have been found to be differentially expressed during phase transition, and one such gene *YPS3 *is induced within 2 h following the 25°C-to-37°C temperature shift [13]. This yeast-phase-specific gene encodes the Yps3p protein that is localized to its cell wall and is also expressed as a secretory protein in infected cells [33,34]. It has been proposed that Yps3p may have a regulatory role in fungal transition and may correlate with pathogenicity [13]. Murine T cells recognize components from cell wall and cell membrane extracts of *H. capsulatum *[35], suggesting that fungal wall components are recognized by immune cells. In this study, we show for the first time that *H. capsulatum *cell membrane protein Yps3p triggers TLR2 signaling and leads to the activation of NF-κB in primary microglial cells.

## Methods

### Organism and culture conditions

*H. capsulatum *G217B [ATCC 26032] is a North American isolate of RFLP class 2 which was termed 'high level' in thermotolerance and pathogenicity. The fungus was grown in *Histoplasma*-macrophage medium (HMM) broth [36] in a 5% CO_2_-95% air atmosphere. Experiments were performed with *H. capsulatum *grown as yeast cells at 37°C.

### Cloning, expression, and purification of recombinant fungal proteins

Recombinant Yps3p and H proteins was prepared as described previously [33,37]. For the preparation of crude cell extract, fractionation was done as follows: log-phase yeast cells were pelleted by centrifugation, washed, and resuspended in PBS. They were then disrupted using glass beads in a Mini-Beadbeater-8 (Biospec Products, Bartlesville, OK) at highest setting for three 1 min periods, separated by chilling on ice for 1 min. Beads were removed by low-speed centrifugation and the cell lysate was spun at 15K RPM in a microcentrifuge at 4 C for 30 min. The supernatant was removed as the cytoplasmic fraction. The pellet was resuspended in PBS as the cell wall/membrane fraction.

### Preparation of microglial cultures

Microglial cell cultures were purified from wild-type C57BL/6 and TLR2 KO mice (Jackson Laboratories, Bar Harbor, ME) using a method described previously with minor modifications [38]. Briefly, cerebral cortical cells from 1-d-old mice were dissociated after a 30 min trypsinization [0.25%] and plated in 75-cm^2 ^Falcon culture flask in DMEM (Sigma-Aldrich, St. Louis, MO) containing 10% heat-inactivated FBS (Hyclone Laboratories, Logan, UT)and penicillin/streptomycin (Sigma-Aldrich). The medium was replenished 1 and 4 d after plating. On d 8 of culture, flasks were shaken for 20 min at a speed of 180 rpm in an orbital shaker to remove unattached cells. On d 12 of culture, microglia floating in the media were collected by aspiration, pooled, centrifuged and seeded at appropriate densities after counting. The cells were washed twice with fresh medium 1 h after seeding to remove non-adherent cells. Microglia prepared this way stain 95–98% positive with Mac-1 antibody (Roche Applied Science, Indianapolis, IN).

### Cloning of VV TLR inhibitors

DNA obtained from the VV Western Reserve strain was used to clone four viral gene products: A46R, A52R, N1L and K1L using PCR. Primers used for amplification were: A46R: Forward: 5'-CAT GCC ATG GCG TTT GAT ATC AGT-3' and Reverse: 5'-CAT GCC ATG GAT GGC GTT TGA TAT-3'; A52R: Forward: 5'-CAT GCC ATG GAC ATA AAG ATA GAT-3' and Reverse: 5'-GTG GAA ATG TCA TAG GCT AGC TAG-3'; N1L: Forward: 5'-CAG GTC ATG AGG ACT CTA CTT ATT-3' and Reverse: 5'-CTA GCT AGC TTA TTT TTC ACC ATA-3'; K1L: Forward: 5'-CAG GAT ATC ATG GAT CTG TCA CGA-3' and Reverse: 5'-CTA GCT AGC TTA GTT TTT CTT TAC AC-3'. PCR was performed on a Gradient 40 Robocycler (Stratagene, La Jolla, CA) using *Pfu *polymerase (Stratagene) with the following conditions: initial denaturation at 95°C for 2 min 30 sec, followed by 30 cycles of 95°C for 1 min, annealing at 60°C for 1 min and elongation at 72°C for 3 min. Following PCR amplification, viral gene products were purified using a 0.8% agarose gel and were cloned into pORF5-mIL10 (InvivoGen) by replacing the mIL-10 ORF with each VV ORF as described previously [39]. This vector carries the murine IL-10 ORF under the control of a composite binary promoter comprised of the elongation factor 1α (EF-1α) and the 5' untranslated region of the human eukaryotic initiation factor 4 g (eIF-4 g). The expression vectors thus generated were termed pORF5-A46R, pORF5-A52R, pORF5-N1L and pORF5-K1L. Expression of these viral proteins was confirmed using Western blot analysis [39].

### Luciferase assay

HEK293T cells, as well as wild-type and TLR2 KO microglia, were transfected with 1 μg pNiFty2-Luc plasmid (InvivoGen) expressing an NF-κB-driven firefly luciferase reporter gene. FuGene 6 was used for transfection of the 293T-mTLR2 cells. Primary microglia are post-mitotic cells which are extremely difficult to transfect using standard methods. In this study, they were successfully transfected using the mouse macrophage nucleofection kit (Amaxa Biosystems, Gaithersburg, MD) and the program Y-01 on the nucleofector I device (Amaxa). Although the transfection efficiency using nucleofection was still low (<10%), luciferase expression occured only in cells that took up the pNiFty2-Luc plasmid. Following nucleofection, the cells were plated in 12-well plates and incubated overnight at 37°C. To stimulate TLR2 signaling, 0.01% heat-killed *L. monocytogenes *(InvivoGen) was added to the culture medium for 5 h. The cells were then lysed and luciferase activity was measured using Bright-Glo luciferase assay substrate (Promega, Madison, WI) on the IVIS^® ^Imaging System (Xenogen Corporation, Alameda, CA). Expression levels of the luciferase reported gene were quantified using Living Image^® ^software (Xenogen). Tranfection efficiencies were tested using a control plasmid expressing green fluorescent protein under the control of CMV IE promoter and the values were normalized to the transfection efficiencies obtained.

### ELISA assay

A sandwich ELISA-based system was used to quantify CCL2 levels from WT and TLR2 KO murine microglial cell culture supernatants. ELISA plates were coated with rat-anti-mouse CCL2 capture antibodies (R&D Systems, Minneapolis, MN) at 1–2 μg/ml overnight at 4°C. The plates were washed (0.05% Tween-20 in phosphate-buffered saline, PBS) and blocked with 1% BSA in PBS for 1 h at 37°C. Detection antibodies (biotinylated goat anti-mouse CCL2 antibodies, 1–2 μg/ml; R&D Systems) were added for 90 min at room temperature followed by peroxidase conjugated strepavidin (1:3000; Jackson Immunoresearch) for 45 min. A chromogenic substrate (K-blue; Neogen Corporation, Lexington, KY) was then added and color development was stopped with 1 M H_2_SO_4_. Absorbance values at 450 nm were used to quantify chemokine levels based on the standard concentration curve generated from serial dilutions.

## Results

### *Histoplasma capsulatum *protein Yps3p is a ligand for Toll-like receptor 2

**Figure 1:**
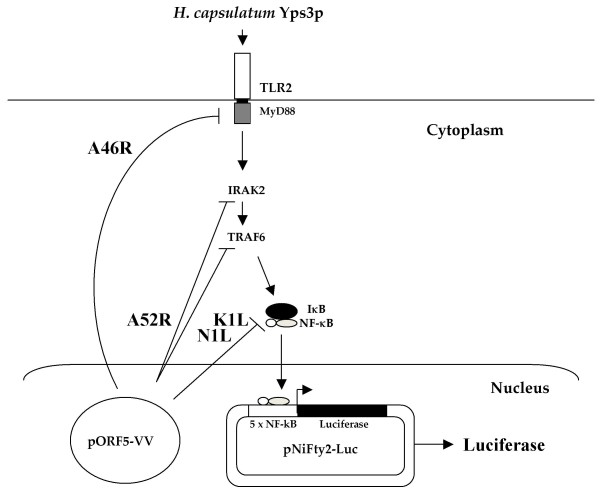
Experimental design. Induction of TLR signaling by *H. capsulatum *Yps3p protein leads to the activation of IRAK2 and NF-κB. NF-κB is then released from its inhibitor IκB and translocates into the nucleus, where it binds to one of five NF-κB binding sites on the pNiFty2-Luc plasmid, and subsequently activates transcription to produce luciferase. Vaccinia virus [VV] protein A46R targets multiple TLR adaptors including MyD88, and A52R associates with IRAK-1 and TRAF6 to disrupt downstream signaling. VV N1L and K1L proteins prevent the release of NF-κB from IκB. Expression of these proteins from their corresponding pORF5-VV plasmid leads to the inhibition of NF-κB activation and decreased luciferase expression from pNiFty2-Luc.

TLRs recognize PAMPs on the surface of pathogens and activate the host's innate immune responses. A number of fungal cell wall components such as mannan, phospholipomannan, and zymosan have previously been shown to be recognized by TLR2 and TLR4 [18,19,29,40]. In order to determine which component of the *H. capsulatum *cell wall/membrane activated the TLR2 signaling pathway, we have used a stable cell line that expresses murine TLR2 under the control of a composite promoter comprised of the eukaryotic elongation factor-1α (EF-1α) core promoter and the R segment, as well as part of the U5 sequence, of the human T-cell leukemia virus type 1 long terminal repeat [39]. These 293T-mTLR2 cells were transfected with the plasmid pNiFty2-Luc, containing the open-reading frame for luciferase under the regulation of five NF-κB binding sites. Using this experimental design, signaling from TLR2 results in the activation of NF-κB which, in turn, activates luciferase expression. Thus, luciferase expression does not occur in the absence of TLR2 signaling. (Fig. 1).

**Figure 2:**
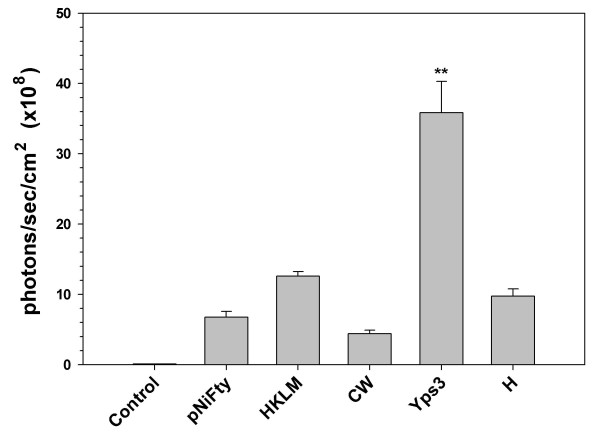
Identification of Yps3p as a TLR2 ligand in 293T cells. pNiFty2-Luc transfected 293T-mTLR2 cells were treated with *H. capsulatum *cell wall/membrane fraction [CW], recombinant Yps3p [Yps3p], and recombinant protein H [H]. Untreated cells were used as a control for background luciferase expression [pNIFty] and heat-killed *Listeria monocytogenes *[HKLM] was added to cells as a positive control for TLR2 signaling. Data are presented as mean ± SD of triplicate samples and are representative of three independent experiments. Statistical analysis was performed by student's *t *test. *P < 0.05; **P < 0.01.

The fungal cell wall fraction was isolated and used to treat 293-mTLR2 cells. Two other fungal proteins Yps3p and H, purified following expression in *E. coli*, were also tested. H protein serves as a negative control and heat-killed *Listeria monocytogenes*, a strong inducer of TLR2 signaling, was used as a positive control. As shown in Fig. 2, neither the cell wall fraction (CW) nor the recombinant H protein activated signaling through TLR2. There was no significant luciferase production above the background levels in these samples. On the other hand, the recombinant protein Yps3p induced TLR2 activation that resulted in a marked increase in luciferase production, demonstrating that Yps3p protein is a ligand for TLR2.

### Vaccinia virus proteins inhibitors of TLR signaling blunt Yps3p-induced luciferase expression

**Figure 3:**
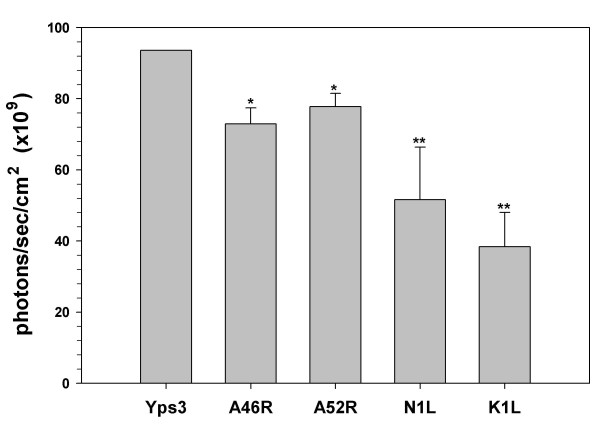
Inhibition of TLR2-mediated, Yps3p-induced NF-κB activation in 293T-mTLR2 cells by VV proteins. Plasmids carrying the open-reading frames of A46R, A52R, K1L, or N1L were co-transfected along with pNiFty2-Luc, cells were incubated overnight at 37°C and treated with Yps3p for 5 h. Cells were then harvested and the amount of luciferase produced was quantified using bright glow substrate. Data are presented as mean ± SD of triplicate samples and are representative of three independent experiments. Statistical analysis was performed by student's *t *test. *P < 0.05; **P < 0.01.

To further confirm the role of TLR2 in responding to *H. capsulatum*, we next attempted to inhibit this signaling pathway using four vaccinia virus([VV) proteins. Among these viral proteins, A46R inhibits signaling from MyD88, the cytoplasmic adaptor of TLR2 [41], and A52R interacts with and blocks the activity of two downstream molecules IRAK2 and TRAF6 along the TLR2 pathway [41,42]; whereas N1L and K1L prevent the release of NF-κB from its inhibitor IκBα [43,44] (Fig. 1). ORFs of each of these VV proteins were cloned into the pORF5 vector under the control of a composite binary promoter comprised of the elongation factor 1α (EF-1α) and the eukaryotic initiation factor 4g (eIF-4g). 1 μg of each pORF5-VV plasmid was co-transfected into 293T-mTLR2 cells together with 1 μg of pNiFty2-Luc. Following overnight incubation at 37°C, the cells were treated with Yps3p for 6 h, harvested and the expression levels of luciferase in the transfected cells were measured using a luciferase assay. The data show that NF-κB activation was severely impaired in cells expressing each of these viral proteins when compared to cells expressing pNiFty2-Luc alone, a result which demonstrates that all four viral proteins were able to inhibit Yps3p-induced TLR2 signaling in 293T-mTLR2 cells (Fig. 3). This result not only confirmed Yps3p mediation of TLR2 signaling but also showed that it can be inhibited at different levels (at the receptor as well as downstream) along the TLR2 signaling pathway.

### TLR2 is required for Yps3p-induced activation of NF-κB in primary murine microglia

**Figure 4:**
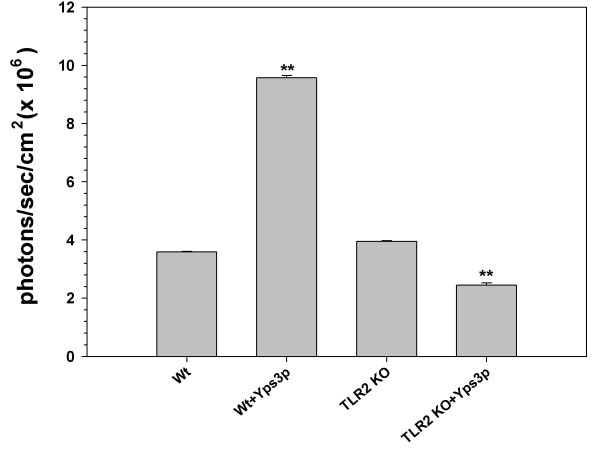
Activation of NF-κB in primary murine microglia following treatment with Yps3p. Purified microglial cells [1 × 10^6^] from wild-type and TLR2 knockout mice were transiently transfected with pNiFty2-Luc plasmid by electroporation using the mouse macrophage nucleofector kit. The cells were incubated overnight at 37°C and 1.5 μg Yps3p was added to the cell culture medium. The microglia were harvested after 5 h and the amount of luciferase produced as a result of NF-κB activation was quantified using the bright glow substrate. Data are presented as mean ± SD of triplicate samples and are representative of three independent experiments. Statistical analysis was performed by student's *t *test. **P < 0.01.

Having determined that *H. capsulatum *protein Yps3p engages TLR2 signaling pathway and causes NF-κB activation in our 293T-mTLR2 cell line, we went on to determine whether Yps3p protein also induced NF-kB activation in primary brain cells. For this experiment, microglial cells from wild-type C57BL/6 mice as well as TLR2 KO mice were isolated and transfected with pNiFty2-Luc plasmid using nucleofection. After overnight incubation at 37°C, the microglia were exposed to the recombinant fungal protein for 6 h. The cells were then harvested and a luciferase assay was performed. In these experiments, Yps3p-induced TLR2 signaling in wild-type microglial cells resulted in high levels of luciferase expression, demonstrating an increased level of NF-κB activation (Fig. 4). In contrast, a significant reduction in luciferase expression occurred following the identical treatment using TLR2 KO microglia. This result further demonstrates that Yps3p triggered signaling through TLR2.

**Figure 5:**
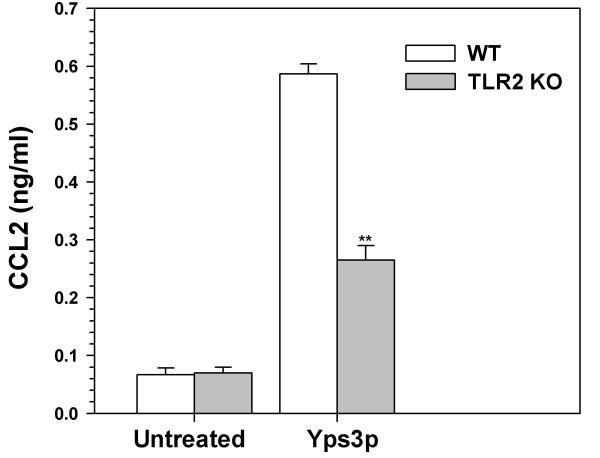
TLR2-mediated expression of CCL2 in response to Yps3p. ELISA assay was performed with purified microglial cells from wild-type and TLR2 knockout mice following exposure with 3 μg/ml Yps3p. Data are presented as relative induction of CCL2 and bars represent the mean ± SD of triplicate samples, which are representative of at least three independent experiments. Statistical analysis was performed by student's *t *test. **P < 0.01.

To further test the role of Yps3p in triggering TLR2 signaling, we performed an ELISA for the proinflammatory immune mediator Chemokine (C-C motif) ligand 2 (CCL2) in wild-type and TLR2 KO microglial cells. The amount to CCL2 secreted into the culture supernatants of microglial cells was quantified using an ELISA assay. While there was no difference in the expression of CCL2 between untreated samples, the expression of CCL2 was elevated in the both wild-type and TLR2 KO microglia following the treatment with Yps3p (Fig. 5). However, the expression levels obtained for TLR2KO microglia were significantly lower than those obtained for wild-type microglia, suggesting that the activation of TLR2 by Yps3p results in the production of CCL2 in these cells.

## Discussion

Airborne invasive fungal pathogens can cause morbidity and mortality in immunocompromised individuals, including those with HIV/AIDS. *H. capsulatum *is a major cause of respiratory infections worldwide and is the etiologic agent of histoplasmosis. In addition to respiratory infections, histoplasmosis has been reported to occur in the brain [1,3-5]. In the present study, we showed that the interaction of *H. capsulatum *Yps3p with microglial cells leads to NF-κB activation via the TLR2 pathway, in both a stable cell line expressing murine TLR2 as well as in primary microglia.

Studies aimed at understanding the role of TLRs in fungal recognition have been controversial. While TLR2 has been shown to be essential for immune responses in macrophages [28], TLR2 KO mice were found to be resistant to candidiasis. It has been reported that both TLR2 and TLR4 are key cellular receptors that recognize opportunistic fungal pathogens such as *C. albicans*, *A. fumigatus *and *C. neoformans*. Phosholipomannan, a unique glycoprotein in the cell wall of *C. albicans*, is a ligand of TLR2, and when mouse macrophages are infected with *C. albicans *they activate NF-κB and produce TNF-α. [28,40]. While TLR2 has been shown to be essential for defense against *C. albicans *[38], contrasting results were reported with TLR2 KO mice being resistant to candidiasis whereas TLR4 KO mice were susceptible [26,27]. Both *in vitro *experiments using macrophages and transfected cell lines, as well as *in vivo *experiments with experiments using TLR-deficient mice infected with *A. fumigatus*, have suggested a role for TLR2 and TLR4 [23,25,31,45]. Similarly, one study showed that TLR2 signaling was necessary for host defense against *C. neoformans *[24], while another reported limited involvement of TLR2 and TLR4 in response to *C. neoformans *infection [32].

## Conclusion

In this study, we report for the first time that *H. capsulatum *triggers TLR2 signaling leading to NF-κB activation in microglial cells and that Yps3p protein is an important fungal component that induces TLR2 signaling. A deeper understanding of host-pathogen interactions will enable us to tackle new challenges posed by fungal pathogens and develop improved therapeutic measures to treat histoplasmosis as well as other deadly mycological diseases.

## Competing interests

The authors declare that they have no competing interests.

## Authors' contributions

RNA and JRL conceived the study. RNA and SH performed experiments. JPW provided *H. capsulatum *materials. RNA and JRL analyzed the data. RNA designed the study and drafted the manuscript. All authors have read and approved the final manuscript.
